# Determinants of access to hemodialysis services in a metropolitan region of Brazil

**DOI:** 10.1186/s12889-022-14258-7

**Published:** 2022-10-07

**Authors:** Ana Cristina de Oliveira Soares, Monica Cattafesta, Mirian Patrícia Castro Pereira Paixão, Edson Theodoro dos Santos Neto, Luciane Bresciani Salaroli

**Affiliations:** 1 Graduate Program in Public Health of Federal University of Espirito Santo (UFES), Health Science Center, Federal University of Espirito Santo (UFES), Av. Marechal Campos, 1468 - Bonfim, Vitória, ES CEP 29047–105 Brazil; 2 Salesiano University Center (UniSales), Av. Vitória, 950 - Forte São João, Vitória, ES 29017-950 Brazil

**Keywords:** Health services accessibility, Hemodialysis, Chronic kidney disease

## Abstract

**Introduction:**

The increasing prevalence of chronic kidney disease has made it a public health issue. Research on access to hemodialysis services is fundamental for appropriate and assertive approaches to the disease. This study analyzed the factors that influence access to hemodialysis services, from the dimensions of availability, accessibility, and acceptability.

**Methods:**

This was a cross-sectional census epidemiological study involving 1024 individuals in the Metropolitan Region of Brazil in 2019. Data were analyzed using multinomial logistic regression.

**Results:**

Factors that increase the chance of belonging to the lowest level of access were: age group from 30 to 59 years (OR 2.16, IC_95%_ 1.377–3.383), female (OR 1.74, IC_95%_ 1.11–2.72), and lower income or equal to two minimum wages (OR 1.80, IC_95%_ 1.17–2.76); the factors medium coverage of the family health strategy or the gateway to public health policy in Brazil (OR 0.54, 95%CI 0.29–0.99), no previous conservative treatment (OR 0.59, 95%CI 0.38–0.91), lack of paid work (OR 0.35, 95%CI 0.15–0.85), retirement/sick leave (OR 0.27, 95%CI 0.12–0.64), and self-assessment of health status as bad or very bad (OR 0.62, 95%CI 0.40–0.96) reduced the chance of belonging to the lowest access level.

**Conclusion:**

Access to hemodialysis services in a metropolis in the southeastern region of Brazil is influenced by contextual, predisposing, enabling, and health needs characteristics. Those who are female, aged between 30 and 59 years, having an income less than or equal to 2 times minimum wage in Brazil, are at the lowest levels of access, which reinforces the role social determinants in health.

## Introduction

Chronic kidney disease (CKD) is a public health problem due to its increasing prevalence and its association with population aging, as well as untreated/controlled conditions of other non-communicable chronic diseases (NCDs), such as diabetes mellitus (DM) and systemic arterial hypertension (SAH) [1, 2]. Social inequalities in health have also been reported as determinants for the development of CKD [2, 3]. The estimated prevalence of CKD in developed countries ranges from 10 to 13% of the adult population, whereas in underdeveloped countries these data are still uncertain [4, 5]. In Brazil, a systematic review on self-reported health status indicated that the prevalence of CKD is around 1.4% of the adult population, although according to the authors themselves, this number may be underestimated [6]. Neves et al. [7] noted that among individuals with CKD in Brazil in 2018, more than 133,000 underwent treatment with renal replacement therapy (RRT), representing an increase of 58% in the period from 2009 to 2018. In addition, more than 92% underwent hemodialysis treatment. Jesus et al. [8] evaluated quality of life in individuals undergoing hemodialysis in Brazil and found that, compared with the control group, people who underwent hemodialysis on a regular basis have lower scores in the physical and psychological domains. It is noteworthy that RRT has a multidimensional approach and depends on conditions of access to health services [9, 10]. According to estimates by the Global Burden of Disease [11], more than 2 million people with CKD worldwide died in 2010 due to lack of access to health services.

The approach to access to health services in the scientific literature has evolved over the years, adding a strong historical component [12–14]. More recently, investigations on the subject have covered the perspectives proposed by McIntyre and Mooney [12] and Thiede and McIntyre [13], which incorporate individual attributes that affect users’ ability to access health services. These authors describe four dimensions that relate to the concept of access in the scope of health services: availability, acceptability, ability to pay (accessibility), and information. They also reinforce aspects of information asymmetry present among the actors involved in the process of access to health [13], while Andersen [14] proposed that access to health services is affected by contextual, enabling, predisposing, and health needs characteristics that can be applied to CKD patients.

In Brazil, the topic has been studied based on international constructs, analyzing aspects of inequality within the context of the country’s public policy [15], which has guaranteed universal access to health since the constitution of 1988 [16]. However, despite this constitutional guarantee, there are still difficulties and barriers in the implementation of access [17], especially for services of high complexity [7, 10, 18] such as hemodialysis. Although there has been a specific public policy in the country since 2004 for individuals with CKD [19], the implementation of this line of care only began in 2014, and data on access to hemodialysis services are still poorly known [4, 20]. Research on the topic has generally only addressed the cost-effectiveness and/or bottlenecks in the supply of health services and/or information [21–23], even considering the growth in demand and the increase in costs of these services, especially in Brazil [22–24].

This study thus presents an unprecedented and innovative proposal in the evaluation of the determining factors of the access of patients with CKD to hemodialysis services, by using the theoretical concepts about access proposed by Thiede et al. [13], systematized within the Behavioral Model of Use of Andersen Health Services [14]. In view of these considerations, this study analyzed the determinants of access to hemodialysis services in a metropolis in southeastern Brazil to provide information to support planning, actions, and health policies to assist patients with CKD.

## Methods

This was a cross-sectional epidemiological census that considered a total of 1351 users who underwent hemodialysis in the studied metropolis in 2019.

This study was carried out in all hemodialysis units that treated patients with chronic kidney disease at the metropolitan region in the Espirito Santo’s, Brazil, at the time of data collection. Of the 1351 users of hemodialysis clinics, 304 were excluded because they met the exclusion criteria (137 were in contact precautions, 74 were hospitalized, 40 had mental confusion, 19 had severe communication impairments, and 34 were very debilitated or had serious physical difficulties). The remaining 1047 participants who met the inclusion criteria were invited to participate in the research. Of these, only 23 people (2.2%) refused to participate.

The inclusion criteria were being over 18 years of age, undergoing HD treatment at the metropolitan region in the Espirito Santo’s capital (state located in the southeastern region of Brazil), being ambulatory, and having a diagnosis confirmed in the medical record of CKD according to the International Classification of Diseases, version 10 (ICD-10), namely, ICD 10: N18 (chronic renal failure); ICD 10: N180 (end-stage renal disease); ICD 10: N188 (other chronic renal failure); ICD 10: N189 (chronic renal failure unspecified); or ICD 10: N19 (renal failure unspecified).

As exclusion criteria included individuals in contact precautions, those who were hospitalized, those with speech and/or hearing impairment, individuals who were debilitated and/or with physical difficulties, and those transferred for hemodialysis to clinics located outside the metropolitan region of the metropolis, in addition to individuals who had ascites. Of the total number of individuals in the target population, 304 were within the exclusion criteria, so the number of eligible individuals for research was 1047. All eligible individuals were invited to participate in the study and of these, 23 (2.2%) refused; the final sample thus consisted of 1024 individuals.

Access was evaluated according to the theoretical propositions of Thiede et al. [13], according to the availability of services, the ability to pay, and acceptability.

The availability dimension is related to the existence of health services that meet the demands of users at the time and place they are needed, reflecting the space-time adjustment between the health needs of individuals and the services offered by the health system. Thus, aspects such as physical and geographic distance between the individual’s home and health services, opening hours of services, and the availability of transport for health professionals to meet emergency demands are included in this dimension.

The ability to pay (accessibility) dimension refers to the adjustment between the direct and indirect costs of the health services demanded and the individual’s financial capacity to assume them, also involving the individual condition of mobilizing financial/economic resources, if necessary. Although in some contexts there is universal health coverage that reduces the asymmetry in the adjustment, expenses related to transportation, food, medication, and even absence from work activities due to a health condition must be analyzed, as they are included in this dimension.

The acceptability dimension, meanwhile, refers to more subjective aspects involved in the relationships between service users and the professionals who provide these services within the health system, highlighting ethical perceptions in these relationships, such as individual, cultural, social, ethnic, and individual respect as a possibility of dialogue in a health professional × service user relationship, based on the perception of mutual respect [13].

To analyze the access data, a judgment matrix adapted from Wilkinson, Warmucci, and Noureddine [25] and Rose et al. [26] was used to construct a score for each of the three access dimensions (availability, ability to pay, and acceptability).

The sum in each dimension was interpreted considering categories for three levels of access according to tertile: the 1st tertile represents the lowest level of access, the 2nd tertile represents the second (intermediate) level of access, and the 3rd tertile represents the highest level of access.

The independent variables were defined from the fifth phase of the Behavioral Model for the Use of Health Services by Andersen [14], which proposes an explanatory model for the use of services based on contextual characteristics related to the socio-geographical environmental environment in which the individual is located, as well as aspects related to the degree of social economic development that affects the living condition (contextual characteristics of the individual’s municipality of residence: Average Human Development Index [IDHM], 2010; GINI index, 2010; Social Vulnerability Index [IVS], 2013; Primary Health Care [PHC] coverage, 2018; Family Health Strategy Coverage [ESF; the gateway to public health policy in Brazil], 2018 [these last two, Brazilian policies, adopted as a gateway to the public health system]; and Mortality Index General, 2019).

The characteristics related to socioeconomic and cultural conditions in an individual dimension, which affect the individual’s ability to access services (income in amount relative to current minimum wage, education in complete years of study, profession, type of access to health services in public or private, municipality of residence in relation to the municipality of hemodialysis).

**Fig. 1:**
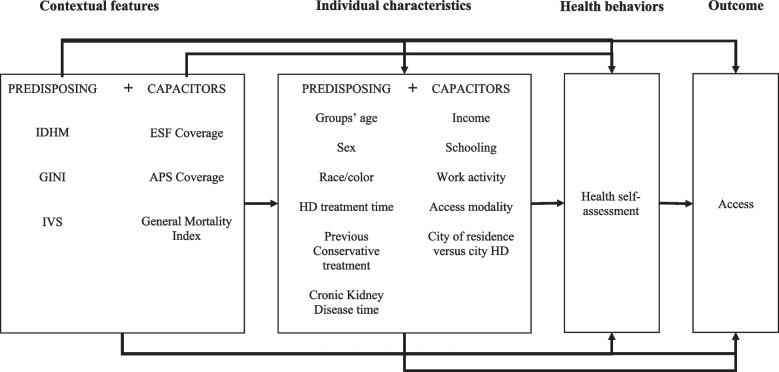
Analysis model. IDHM, Average Human Development Index; GINI, Index used to measure social inequality; IVS, Social vulnerability index; ESF, Family Health Strategy coverage (the gateway to public health police in Brazil); APS, Primary Health Care (Public health police in Brazil); HD, Hemodialysis

Predisposing characteristics related to individual physical/physiological conditions that affect access to health services (age group, sex, race/color, time on CKD, time on hemodialysis, previous conservative treatment); and health need characteristics (individual’s self-assessment of their own health condition as good/very good and bad/very bad), as shown in Fig. 1.

Data collection was carried out using a previously developed and tested instrument and software developed specifically for this collection, to avoid possible failures in the transcription of forms and to optimize the time for collecting research data. The information for the study variables was based on data on the hemodialysis characteristics transcribed from the medical records and/or provided by the individuals; information for the individual variables was contained in the interview questionnaires and on the social indicators as disclosed by the IBGE (2010). Data were analyzed using IBM SPSS Statistics for Windows, version 22.0 (Armonk, NY: IBM Corp).

To assess the reproducibility of the data collection instrument, a pilot test was carried out between October and December 2018, with 57 individuals with renal failure undergoing hemodialysis in a municipality outside the metropolis to be analyzed (not included in the study sample). The instrument, composed of 51 questions divided into three blocks (availability, accessibility/payment capacity, and acceptability) according to Thiede et al. [12], was tested using the software WinPepi for Windows® version 11.65 according to Kappa, adjusted Kappa and McNemar values, with their values (0.78 to 0.98 of agreement and non-significant disagreement) adequate for all variables in the instrument’s dimensions.

Bivariate analyses between access tertiles and user characteristics (contextual, predisposing, enabling, and health needs) were performed using the chi-square test (χ^2^). Multinomial logistic regression analysis was performed to estimate the association of independent variables with the outcome (level of access). For this, variables that presented *p*-values up to 0.1 in the association analyses were included. To build the final regression model, the variables were entered into a model considering the dimensions (contextual, predisposing, enabling, and health needs; see Fig. 1), and only the variables that remained associated with the outcome (*p* < 0.1) were included in the subsequent models.

In the final model (model 3), only the variables that presented *p*-values below 0.05 remained. The confidence interval was 95%. It is noteworthy that only hemodialysis users with responses to all variables were included in this analysis. Furthermore, multicollinearity tests were performed (tolerance > 0.1 and variance inflation factor < 10) and, when they existed (block of contextual variables), we opted to use the most frequently used in the literature. We also determined the minimum sample size for the number of model variables (> 20 individuals per model variable and > 5 cases in each category of variables), the absence of outliers (absence of standardized residues > ± 3 standard deviations; up to 1% of standardized residues between ±2.5 and 3 standard deviations; and up to 5% of standardized residues between ±2.0 and 2.5 standard deviations, Cook’s distance < 1, and DFBeta < 1), and adjustment according to the Model Fitting Information (p-valor < 0.05), Godness-of-Fit (*p*-value > 0.05) and the Nagelkerke test value (0.287).

This study followed all the ethical precepts of the Declaration of Helsinki and was approved by the Research Ethics Committee of the Health Sciences Center of the Federal University of Espírito Santo, under protocol number 4,023,221 (CAAE no. 68528817.4.0000.5060). All hemodialysis units formally authorized the research by signing the letter of consent, and all research participants signed the Informed Consent Term.

## Results

Of the total of 1351 users who underwent hemodialysis during the study period, data were collected from 1024 individuals (75.8%). The mean age was 54.7 + 0.59 years and the predominant age group was between 30 and 59 years (*n* = 528, 51.6%). Most individuals were male (*n* = 581, 56.7%), with up to 8 years of schooling (*n* = 523, 51.6%), self-declared brown/black (*n* = 737, 72%), income less than or equal to two times the minimum wage (*n* = 555, 56.2%), retired or away from work, receiving social benefits (*n* = 547, 54.2%), and residing in the same city where they were undergoing hemodialysis (*n* = 642, 62.8%) (Table 1).

**Table 1:** Descriptive analysis of sociodemographic variables of hemodialysis service users

Variable	n	%
**Age group** (*n* = 1024)		
20–29 years	59	5.7
30–59 years	528	51.6
60 years and more	437	42.7
**Sex** (*n* = 1024)		
Female	443	43.3
Male	581	56.7
**Schooling** (*n* = 1022)		
≤ 8 years of study	523	51.6
> 8 ≤ 11 years of study	332	32.8
> 11 years of study	158	15.6
**Race/color** (*n* = 1016)		
White	274	26.8
Black/Brown	737	72.0
Others	12	1.2
**Income** (*n* = 988)		
≤ 2 minimum wage	555	56.2
> 2 minimum wage	433	43.8
**Profession** (*n* = 1009)		
With paid work activity	348	34.5
Retired due to illness	547	54.2
Without paid work activity	114	11.3
**City of residence and treatment** (*n* = 1023)		
Live in the same city where he/she undergoes treatment	642	62.8
Don’t live in the same city where he/she undergoes treatment	381	37.2
**Acess level** (*n* = 830)		
Lowest level of acess (1° tercile)	281	33.9
Intermediate level of acess (2° tercile)	340	41.0
Highest level of acess (3° tercile)	209	25.1

For the bivariate and multivariate analyses, only the results of individuals who had responses to all variables were considered, so data from 830 individuals were included. Regarding the level of access, 281 individuals (33.9%) were at the lowest level of access, 340 individuals (41%) at the intermediate level of access, and 209 individuals (25.1%) were at the highest level of access (Table 2).

**Table 2:** Descriptive analysis of access dimensions variables (availability, accessibility and acceptability) of hemodialysis service users

Variable	N	%
**Availability**		
**Distance from the home hemodialysis center** (*n* = 1018)		
Less de 1 km	44	4.3
In between 1 a 4 km	226	22.2
In between 5 a 14 km	432	42.4
Over of 15 km	316	31
**Need transport to go to hemodialysis service** (*n* = 1024)		
Yes	946	92.4
Not	78	7.6
**Transport used to go to the hemodialysis service** (*n* = 1021)		
Motorized	992	97.2
Outros (bike for example)	11	1.1
On foot	18	1.8
**Time in transport** (*n* = 1011)		
Equal to or greater than 60 minutes	195	19.3
Between 30 and 59 minutes	296	29.3
Less than 30 minutes	520	51.4
**Public system provides transportation** (*n* = 983)		
Not	204	19.9
Yes	779	76.1
**There is transport for healthcare professionals** (*n* = 896)		
Not	826	92.2
Yes	70	7.8
**Considers the quality hemodialysis service** (*n* = 1023)		
Not	81	7.9
Yes	942	92.1
**Hemodialysis services are what you need** (*n* = 1020)		
Not	48	4.7
Yes	972	95.3
**Accessibility (payment ability)**		
**paid for hemodialysis services** (*n* = 1023)		
Yes	116	11.3
Not	907	88.7
**Needed to buy medicine** (*n* = 1024)		
Yes	689	67.3
Not	335	32.7
**Paid transportation to go to hemodialysis** (*n* = 1018)		
Yes	471	46.3
Not	547	53.7
**Paid for food on hemodialysis** (*n* = 1023)		
Yes	326	31.8
Not	697	68.1
**Missed a day of work to undergo hemodialysis** (*n* = 921)		
Yes	69	7.5
Not	852	92.5
**Lost of financial gains due to hemodialysis** (*n* = 979)		
Yes	248	25.3
Not	731	74.7
**Needed a financial loan with family members** (*n* = 1020)		
Yes	149	14.6
Not	871	85.4
**Needed financial loan with neighbors/friends** (*n* = 1023)		
Yes	75	7.3
Not	948	92.7
**Needed a financial loan with banks** (*n* = 1023)		
Yes	978	95.6
Not	45	4.4
**Needed to sell assets to undergo hemodialysis** (*n* = 1024)		
Yes	30	2.9
Not	994	97.1
**Acceptability**		
**Trust the service professionals** (*n* = 1022)		
Not	69	6.8
Yes	953	93.2
**Receives respectful treatment by professionals** (*n* = 1023)		
Not	24	2.3
Yes	999	97.7
**Agrees with the treatment given** (*n* = 1021)		
Not	49	4.8
Yes	972	94.9
**Your complaints are heard by professionals** (*n* = 1020)		
Not	96	9.4
Yes	924	90.6
**Receive information about alternative treatments** (*n* = 1021)		
Not	341	33.4
Yes	680	66.6
**The service meets your physical needs** (*n* = 1023)		
Not	74	7.2
Yes	949	92.8
**Do you feel some kind of prejudice on the part of professionals** (*n* = 1021)		
Not	933	91.4
Yes	88	8.6
**The service has equipment/devices available to serve you** (*n* = 1019)		
Not	127	12.5
Yes	892	87.4
**The team is trained to serve you** (*n* = 1020)		
Not	50	4.9
Yes	970	95.1
**Believes that it is easy to follow up on health in the public network outside of hemodialysis** (*n* = 1008)		
Not	302	30
Yes	706	70
**Feel free to make any kind of complaint** (*n* = 1006)		
Not	303	30.1
Yes	703	69.9
**Availability**		
**Distance from the home hemodialysis center** (*n* = 1018)		
Less de 1 km	44	4.3
In between 1 a 4 km	226	22.2
In between 5 a 14 km	432	42.4
Over of 15 km	316	31
**Need transport to go to hemodialysis service** (*n* = 1024)		
Yes	946	92.4
Not	78	7.6
**Transport used to go to the hemodialysis service** (*n* = 1021)		
Motorized	992	97.2
Outros (bike for example)	11	1.1
On foot	18	1.8
**Time in transport** (*n* = 1011)		
Equal to or greater than 60 minutes	195	19.3
Between 30 and 59 minutes	296	29.3
Less than 30 minutes	520	51.4
**Public system provides transportation** (*n* = 983)		
Not	204	19.9
Yes	779	76.1
**There is transport for healthcare professionals** (*n* = 896)		
Not	826	92.2
Yes	70	7.8
**Considers the quality hemodialysis service** (*n* = 1023)		
Not	81	7.9
Yes	942	92.1
**Hemodialysis services are what you need** (*n* = 1020)		
Not	48	4.7
Yes	972	95.3
**Accessibility (payment ability)**		
**paid for hemodialysis services** (*n* = 1023)		
Yes	116	11.3
Not	907	88.7
**Needed to buy medicine** (*n* = 1024)		
Yes	689	67.3
Not	335	32.7
**Paid transportation to go to hemodialysis** (*n* = 1018)		
Yes	471	46.3
Not	547	53.7
**Paid for food on hemodialysis** (*n* = 1023)		
Yes	326	31.8
Not	697	68.1
**Missed a day of work to undergo hemodialysis** (*n* = 921)		
Yes	69	7.5
Not	852	92.5
**Lost of financial gains due to hemodialysis** (*n* = 979)		
Yes	248	25.3
Not	731	74.7
**Needed a financial loan with family members** (*n* = 1020)		
Yes	149	14.6
Not	871	85.4
**Needed financial loan with neighbors/friends** (*n* = 1023)		
Yes	75	7.3
Not	948	92.7
**Needed a financial loan with banks** (*n* = 1023)		
Yes	978	95.6
Not	45	4.4
**Needed to sell assets to undergo hemodialysis** (*n* = 1024)		
Yes	30	2.9
Not	994	97.1
**Acceptability**		
**Trust the service professionals** (*n* = 1022)		
Not	69	6.8
Yes	953	93.2
**Receives respectful treatment by professionals** (*n* = 1023)		
Not	24	2.3
Yes	999	97.7
**Agrees with the treatment given** (*n* = 1021)		
Not	49	4.8
Yes	972	94.9
**Your complaints are heard by professionals** (*n* = 1020)		
Not	96	9.4
Yes	924	90.6
**Receive information about alternative treatments** (*n* = 1021)		
Not	341	33.4
Yes	680	66.6
**The service meets your physical needs** (*n* = 1023)		
Not	74	7.2
Yes	949	92.8
**Do you feel some kind of prejudice on the part of professionals** (*n* = 1021)		
Not	933	91.4
Yes	88	8.6
**The service has equipment/devices available to serve you** (*n* = 1019)		
Not	127	12.5
Yes	892	87.4
**The team is trained to serve you** (*n* = 1020)		
Not	50	4.9
Yes	970	95.1
**Believes that it is easy to follow up on health in the public network outside of hemodialysis** (*n* = 1008)		
Not	302	30
Yes	706	70
**Feel free to make any kind of complaint** (*n* = 1006)		
Not	303	30.1
Yes	703	69.9

There was no difference between the levels of access and PHC coverage; however, for the other contextual variables, residing in municipalities with MHDI classified as high and very high (*p* < 0.001), low and very low regions social vulnerability (*p* < 0.001), as well as in municipalities with a lower overall mortality rate (*p* < 0.001), were associated with a higher level of access, while residing in municipalities with a higher concentration of income was associated with lower levels of access (*p* < 0.001). In relation to ESF coverage, there was an association between lower ESF coverage in the municipality of residence and the lowest level of access (*p* < 0.001) (Table 3).

**Table 3:** Distribution of access levels, according to contextual, predisposing, enabling and health needs of hemodialysis service users

Variables	Access levels								
	Total		Low (***n*** = 281)		Intermediate (***n*** = 340)		High (***n*** = 209)		***P*** value*
	n	%	n	%	n	%	n	%	
**Contextual**									
**IDHM** (*n* = 830)									**< 0.001**
Medium	42	5.1	23	8.2	17	5.0	2	1.0	
High	494	59.5	175	62.3	212	62.4	107	51.2	
Very High	294	35.4	83	29.5	111	32.6	100	47.8	
**Gini Index** (*n* = 830)									**< 0.001**
Average income concentration	437	52.7	169	60.1	189	55.6	79	37.8	
High concentration of income	236	28.4	75	26.7	94	27.6	67	32.1	
Very high concentration of income	157	18.9	37	13.2	57	16.8	63	30.1	
**IVS** (*n* = 830)									**< 0.001**
Very low	163	19.6	41	14.6	59	17.4	63	30.1	
Low	259	31.2	85	30.2	107	31.5	67	32.1	
Average	408	49.2	155	55.2	174	51.1	79	37.8	
**Primary health care coverage** (*n* = 830)									**0.603**
High	376	45.3	124	44.2	150	44.1	102	48.8	
Medium	284	34.2	101	35.9	113	33.2	70	33.5	
Low	170	20.5	56	19.9	77	22.7	37	17.7	
**Family health strategy coverage** (*n* = 830)									**< 0.001**
High	213	25.7	83	29.5	91	26.8	39	18.7	
Medium	228	27.5	52	18.5	83	24.4	93	44.5	
Low	389	46.8	146	52.0	166	48.8	77	36.8	
**General Mortality Index** (*n* = 830)									**< 0.001**
Up to 5/1000 inhabitants	314	37.8	107	38.1	138	40.6	69	33.0	
From 5.1 to 10/1000 inhabitants	359	43.3	137	48.8	145	42.6	77	36.9	
Above 10/1000 inhabitants	157	18.9	37	13.1	57	16.8	63	30.1	
**Predisposing**									
**Age group** (*n* = 830)									**< 0.001**
20 to 29 years	47	4.6	18	6.4	19	5.6	10	4.8	
30 to 59 years	446	53.7	185	65.8	165	48.5	96	45.9	
60 and over	337	41.7	78	27.8	156	45.9	103	49.3	
**Race/color** (*n* = 820)									**0.108**
White	227	27.7	67	24.2	102	30.3	58	28.2	
Black	202	24.6	61	22.0	82	24.3	59	28.6	
Brown	391	47.7	149	53.8	153	45.4	89	43.2	
**Sex** (*n* = 830)									**< 0.001**
Female	354	42.7	142	50.5	142	41.8	70	33.5	
Male	476	57.3	139	49.5	198	58.2	139	66.5	
**Chronic kidney disease time** (*n* = 825)									**0.041**
< 5 years	426	51.6	127	45.5	187	55.2	112	54.1	
≥ 5 years	399	48.4	152	54.5	152	44.8	95	45.9	
**Previous conservative treatment** (*n* = 826)									**0.191**
Not	553	66.9	176	63.1	230	67.8	147	70.7	
Yes	273	33.1	103	36.9	109	32.2	61	29.3	
**Hemodialysis treatment time** (*n* = 777)									**0.033**
0 to 2 years	297	38.2	97	36.9	128	40.6	69	34.7	
3 to 5 years	200	25.7	52	19.8	88	27.9	60	30.2	
6 to 10 years	156	20.0	60	22.8	54	17.1	42	21.1	
Above to 10 years	127	16.1	54	20.5	45	14.4	28	14.0	
**Capacitors**									
**Income** (*n* = 806)									**0.002**
≤ 2 minimum wage	461	57.2	173	62.9	192	58.7	96	47.1	
> 2 minimum wage	345	42.8	102	37.1	135	41.3	108	52.9	
**Access modality to hemodialysis services** (*n* = 829)									**0.046**
Health Unic System (Public police)	638	77.0	219	78.2	271	79.8	148	70.8	
Others	191	23.0	61	21.8	69	20.2	61	29.2	
**Schooling** (*n* = 820)									**0.033**
≤ 8 years of study	418	51	151	18.4	181	22.1	86	10.5	
> 8 ≤ 11 years of study	281	34.3	85	10.4	108	13.2	88	10.7	
> 11 years of study	121	14.8	41	5	50	6.1	30	3.7	
**Profession** (*n* = 819)									**< 0.001**
With paid work activity	297	36.3	107	38.8	110	32.8	80	38.5	
Retired due to illness	427	52.1	123	44.6	186	55.5	118	56.7	
Without paid work activity	95	11.6	46	16.7	39	11.6	10	4.8	
**City of residence x hemodialysis treatment city** (*n* = 830)									**< 0.001**
Lives in the same city where he/she undergoes hemodialysis	510	61.4	129	45.9	206	60.6	175	83.7	
Doesn’t live in the same city where he/she undergoes hemodialysis	320	38.6	152	54.1	134	39.4	34	16.3	
**Health needs**									
**Self-assessment of health status** (*n* = 828)									**0.032**
Good /very good	525	63.4	161	57.7	220	64.7	144	68.9	
Bad / Very bad	303	36.6	118	42.3	120	35.3	65	31.1	

Chi-square test. In the descriptive analysis, all individuals with information on each of the variables were included, while in the regression analysis, only those with information available for the set of variables were included. Caption: IDHM: Brazilian Municipal Human Development Index; IVS: Social Vulnerability Index; Primary Health Care; Family Health Strategy; *CKD* Chronic Kidney Disease; *HD* Hemodialysis; SUS: Unified Health System

Related to the predisposing characteristics, belonging to the age group of 60 years and over (*p* < 0.001), being male (*p* < 0.001), having less than 5 years of CKD (*p* = 0.041), and having less than 2 years of hemodialysis treatment (*p* = 0.030) were associated with a higher level of access (Table 3).

Evaluating the enabling characteristics, having an income equal to or less than two times the minimum wage (*p* = 0.002), having 8 years of schooling or less (*p* = 0.033), and not residing in the same city where hemodialysis procedures are performed (*p* < 0.001) were associated with the lowest level of access; while accessing hemodialysis services through the Unified Health System (SUS) (*p* = 0.046), having paid work, and receiving social benefits (*p* < 0.001) were associated with a higher level of access. Related to health needs, self-assessment of the health condition as good/very good (*p* = 0.032) was associated with the highest level of access (Table 3).

The results of the multinomial logistic regression analysis (Table 4) demonstrated that the factors that increased the chances of belonging to the lowest level of access compared to the highest level of access were: being in the age group between 30 and 59 years (95%CI 1.377–3.383; OR 2.16), being female (95%CI 1.11–2.72; OR 1.74), and belonging to an income range less than or equal to two times the minimum wage (95%CI 1.17–2.76; OR 1.80). Having average ESF coverage (95%CI 0.29–0.99; OR 0.54), not undergoing previous conservative treatment (95%CI 0.38–0, 91; OR 0.59), not residing in the same city as hemodialysis treatment (95%CI, 0.08–0.22; OR 0.13), not having a paid job (95%CI 0.15–0.85; OR 0.35), being retired or away from work receiving social benefits (95%CI 0.08–0.22; OR 0.13), and self-assessing the health condition as poor/very poor reduced the chances of belonging to the lowest level of access.

**Table 4:** Final model of the odds ratio of the access level of the first and second tertiles of hemodialysis service users

Variables		Model 1	Model 2	Model 3
		OR (IC_**95%**_)	OR (IC_**95%**_)	OR (IC_**95%**_)
		First tertile (lowest access level)	First tertile (lowest access level)	First tertile (lowest access level)
**Contextual**	**IDHm**			
	Medium	**26.14 (3.61–189.12)**	5.61 (0.85–36.94)	
	High	**10.25 (1.87–56,021)**	6.78 (1.00–44.5)	
	Very High	1	1	
	**General Mortality Index**			
	Up to 5/1000 inhabitants	1	1	
	5,1 to 10/1000 inhabitants	**0.11 (0.19–0.66)**	0.28 (0.39–1.94)	
	Above to 10/1000 inhabitants	**0.09 (0.15–0.58)**	0.18 (0.24–1.36)	
	**Family health strategy coverage**			
	Low Coverage	**0.16 (0.03–0.79)**	0.40 (0.07–2.44)	1.43 (0.63–3.26)
	Medium Coverage	**0.05 (0.01–0.26)**	**0.16 (0.03–0.98)**	**0.54 (0.29–0.99)**
	High Coverage	1	1	1
**Predisposing**	**Age groups**			
	Up to 29 years	2.44 (0.93–6.35)	1.78 (0.62–5.14)	1.73 (0.62–4.83)
	30 to 59 years	**2.25 (1.47–3.48)**	**2.08 (1.30–3.32)**	**2.16 (1.38–3.39)**
	≥ 60 years	1	1	1
	**Sex**			
	Female	**2.10 (1.40–3.17)**	**1.77 (1.13–2.80)**	**1.74 (1.11–2.72)**
	Male	1	1	1
	**Race/color**			
	Black	0.98 (0.60–1.63)		
	Brown	0.75 (0.46–1.22)		
	White	1		
	**Hemodialisys treatment time**			
	0 to 2 years	1.05 (0.47–2.34)		
	3 to 5 years	0.57 (0.27–1.20)		
	6 to 10 years	0.77 (0.40–1.48)		
	Above to 10 years	1		
	**Chronic kidney disease time**			
	< 5 years	0.85 (0.46–1.59)	0.78 (0.51–1.20)	
	> 5 years	1	1	
	**Previous conservative treatment**			
	Not	0.68 (0.44–1.06)	**0.62 (0.39–0.99)**	**0.59 (0.38–0.91)**
	Yes	1	1	1
**Capacitors**	**Income**			
	≤ 2 minimum wage		**1.75 (1.07–2.84)**	**1.80 (1.17–2.78)**
	> 2 minimum wage		1	1
	**Schooling**			
	≤ 8 years of study		1.08 (0.52–2.27)	
	> 8 ≤ 11 years of study		0.75 (0.38–1.50)	
	> 11 years of study		1	
	**Access modality to hemodialysis services**			
	Health Unic System (Public police)		1.14 (0.66–2.00)	
	Others		1	
	**Profession**			
	Without paid work activity		0.44 (0.18–1.07)	**0.35 (0.15–0.85)**
	Retired due to illness		**0.33 (0.14–0.78)**	**0.27 (0.12–0.64)**
	With paid work activity		1	1
	**City of residence x hemodialysis treatment city**			
	Doesn’t live in the same city where he/she undergoes hemodialysis		**0.14 (0.08–0.24)**	**0.13 (0.08–0.22)**
	Lives in the same city where he/she undergoes hemodialysis		1	1
**Health needs**	**Self-assessment of health status**			
	Bad/very bad			**0.62 (0.40–0.96)**
	Good /Very good			1

The First tertile (lowest level of access) was compared to the last tertile (highest level of access). The highest level of access is referral

Values ​​related to the second tertile are not shown in Table 4, as they did not remain significant in any of the regression models

Variables that presented *p*-values up to 0.1 in the association analyses were included. The variables were entered into a model considering the dimensions (contextual, predisposing, enabling, and health needs; see Fig. 1). To build the final regression model, only the variables that were remained associated with the outcome in the previous blocks were included in the subsequent models

## Discussion

The results demonstrate that access to hemodialysis services is multidimensional and involves complex factors related to the contextual, predisposing, enabling, and health need aspects of users of these services. The determining factors for patients with CKD on hemodialysis belonging to the lowest level of access were being in the age group of 30 to 59 years, being female, and having an income of less than or equal to two times the minimum wage. These data reinforce the sense of integrality involved in the issue, as well as portraying the panorama of access to hemodialysis services in the Brazilian metropolis studied.

When evaluating the context in which hemodialysis users are inserted, the findings indicate an association between average ESF coverage and lower chances of belonging to the lowest level of health service access. These data can be interpreted from the logic of the organization of the ESF with population coverage in regions with greater demand for services, combined with lower socioeconomic status, and the establishment of a bond with citizens, thus reducing the distances between the service user and the organized health system. This results in the effectiveness/assertiveness of the care approach, regardless of the level of complexity required [27]. This strategy, when working on health education actions, dissemination of information on sustained self-care, and monitoring of health conditions, becomes fundamental for the establishment of the referral and counter-referral processes within the scope of the SUS in situations that demand high and medium levels of service complexity [28], such as hemodialysis. This fact confirms the propositions of Mendes et al. [29] who highlighted the role of problem-solving services based on the SUS organization logic in health care networks [30].

Most individuals undergoing hemodialysis were male, a result similar to those of other studies [7, 20, 31]. However, the results suggest inequality of access between genders, because women were at the lowest level of access compared to men. Although studies have shown that women tend to seek health services more than men do, such data involve low-complexity services with a focus on prevention [32], while in the present study, the approach was among individuals accessing high-complexity services. This fact is strongly influenced by enabling characteristics and affected by social and gender inequalities. This contrasts with inclusive policies in Brazil, where women still face different more barriers to accessing health services compared to men [33].

The absence of previous conservative treatment for CKD was associated with lower chances of belonging to the lowest level of access. These data can be interpreted based on the severity of the health condition of individuals diagnosed with chronic renal failure already in the RRT indication phase, who are therefore promptly referred to high-complexity health services, which does not necessarily occur with people following up on an outpatient basis in routine elective consultations [4, 18].

It is also noteworthy that the age group between 30 and 59 years was at the lowest level of access, despite the average age of users of hemodialysis services being within this range. This result can be analyzed from the perspective of the logic of economic productivity, because it includes the age group with greater insertion in the country’s labor market [34], reflecting greater difficulty for individuals in this age group to leave work to access health services that demand a weekly routine without the consequences of unemployment; this is an important social concern in underdeveloped and unequal countries such as Brazil.

These data may also be related to the fact that older individuals have a longer period of illness and, consequently, greater acceptance of their health condition, reflecting a higher score in the acceptability dimension and more free time due to not having a job. A similar result was found in other studies, which, although not working with a group of renal patients undergoing hemodialysis, showed the same relationship [30, 32]. The same analysis can be made for data that indicate that not having a paid job and being retired or away from work receiving social benefits reduce the chances of belonging to the lowest level of access to health services. Although the health condition of the population groups were approached in an isolated way, health is an intangible economic asset, as sick individuals in the productive age group, without access to health services, evolve to aggravation of the disease, so they may need early social assistance/benefits, contributing to economic retraction and social poverty, which in turn, negatively affect the health conditions of individuals and the community [35].

The association between low income and the lowest level of access also reinforced the concept of social determinants in health [36–38]. Studies have shown that poorer social groups and, therefore, more socially vulnerable groups, have greater barriers to accessing goods and services, including health services [32, 36]. These findings are similar to those found by other authors [20, 31, 39], and can be analyzed from Marmot [40], who explains the relationship between income and health: this relationship can occur both in the sense that low income leads to worse health conditions, and in the sense that poor health conditions make it difficult for the individual to earn income, which reinforces the role of the social context in determining the health–disease process.

Inequalities can also be evaluated from the perspective of the location of the services offered and the physical/geographical distance from the location of the individuals who demand the services. The result of this study demonstrated, however, that residing in a different municipality from where the hemodialysis center would reduce the chances of belonging to the lowest level of access. This can be interpreted from the resoluteness of the regionalization in health adopted in Brazil’s health policy, with the guarantee of sanitary transport for patients who need to move to other cities, in addition to the commitment to the scheduling of transport by the user, which may not occur with individuals who perform the procedure in the municipality of residence, as well as reflecting the economic difficulty of bearing the cost of public transport, which would not occur in the case of intercity sanitary transport [28, 36, 41]. This result may also reflect the small territorial extension of the studied metropolitan region.

Self-assessment of health status, although a subjective measure, has been reported as a reliable marker of the use of health services, along with mortality and functional disability [42], that is strongly affected by physical health conditions and sociodemographic aspects. A previous study, which, although it did not assess CKD patients undergoing hemodialysis, found that, despite this assessment including subjective aspects, positive self-perception was associated with better effective health conditions, which reinforced the integral dimension of the health concept far beyond the physical condition [43]. In this sense, there is already evidence that patients with chronic diseases, especially those with more than one chronic health condition, tend to assess their own health status more negatively [43, 44]. The results indicating that individuals who self-assess their health condition as bad and very bad compared to those who assess it as good and very good are less likely to be at the lowest level of access, can be explained by the fact that they are carriers of chronic health condition with priority and continuous care and that, in this way, they feel closer to the health system [7].

ased on the findings of the present study, it is possible to contribute to the monitoring of the actions provided for the care line for persons with CKD [45–48], because, based on the methodology adopted, the data reliably portray the reality of access to hemodialysis services in a capital city in southeastern Brazil. Despite this approach, one of the limitations of this study is its cross-sectional nature, which requires greater caution in interpreting the results, due to the possibility of reverse causality. In addition, this study was carried out in a single state in Brazil. However, the methodological designs adopted to circumvent this limitation included evaluating individuals in all hemodialysis clinics in the metropolitan region surveyed, which concentrates the largest number of individuals undergoing hemodialysis in the state and conducting a pilot test to analyze the robustness of the data collection instrument.

## Conclusion

Access to hemodialysis services in a metropolis in the southeastern region of Brazil is influenced by contextual, predisposing, enabling, and health need characteristics. Female health service users, aged between 30 and 59 years, with an income less than or equal to two times the minimum wage in force in Brazil, are at the lowest levels of access.

These data show aspects of inequality in access to hemodialysis services, which reinforces the importance of developing public policies to organize the distribution and supply of services to reduce social inequities, as well as ensure that the SUS is strengthened as a public health system policy, because, although the health policy in Brazil is universal, the data from this study show that there are still inequalities in access to hemodialysis services.

Based on the results presented, PHC as Brazilian public policy seems to be a viable way to expand access and reduce health inequalities by bringing the individual closer to the established health system.

## Data Availability

Dataset used and/or analyzed during the current study are available from the corresponding author upon reasonable request.

## Abbreviations

APS: Primary Health Care (Public health police in Brazil)
CKD: Chronic kidney disease
DM: Diabetes Mellitus
ESF: Family Health Strategy Coverage (the gateway to public health police in Brazil)
IBGE: Brazilian Institute of Geography and Statistics
IDHM: Average Human Development Index
IVS: Social Vulnerability Index
NCDs: Non-Communicable Chronic Diseases
RRT: Renal replacement therapy
SAH: Systemic Arterial Hypertension
